# Multimodal pain assessment improves discrimination between noxious and non‐noxious stimuli in infants

**DOI:** 10.1002/pne2.12007

**Published:** 2019-09-09

**Authors:** Marianne van der Vaart, Eugene Duff, Nader Raafat, Richard Rogers, Caroline Hartley, Rebeccah Slater

**Affiliations:** ^1^ Department of Paediatrics University of Oxford Oxford UK; ^2^ Nuffield Department of Anaesthesia John Radcliffe Hospital Oxford UK

**Keywords:** EEG, infant, machine learning, pain

## Abstract

Infants in neonatal intensive care units frequently experience clinically necessary painful procedures, which elicit a range of behavioral, physiological, and neurophysiological responses. However, the measurement of pain in this population is a challenge and no gold standard exists. The aim of this study was to investigate how noxious‐evoked changes in facial expression, reflex withdrawal, brain activity, heart rate, and oxygen saturation are related and to examine their accuracy in discriminating between noxious and non‐noxious stimuli. In 109 infants who received a clinically required heel lance and a control non‐noxious stimulus, we investigated whether combining responses across each modality, or including multiple measures from within each modality improves our ability to discriminate the noxious and non‐noxious stimuli. A random forest algorithm was used to build data‐driven models to discriminate between the noxious and non‐noxious stimuli in a training set which were then validated in a test set of independent infants. Measures within each modality were highly correlated, while different modalities showed less association. The model combining information across all modalities had good discriminative ability (accuracy of 0.81 in identifying noxious and non‐noxious stimuli), which was higher than the discriminative power of the models built from individual modalities. This demonstrates the importance of including multiple modalities in the assessment of infant pain.

## Graphical abstract

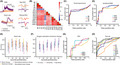

## 1 INTRODUCTION

Pain exposure during early life not only causes acute distress, but may also have long‐term neurodevelopment consequences,^1^, ^2^, ^3^, ^4^ leading to the urgent need to improve pain management and treatment in infants. However, due to the subjective nature of pain, its assessment in nonverbal infants is challenging and must rely on surrogate measures.^5^ Noxious stimulation elicits a wide range of responses mediated at different levels of the nervous system, including intensity graded reflex withdrawal of both the ipsilateral and contralateral limb,^6^, ^7^, ^8^ physiological changes 9 such as increases in heart rate^10^, ^11^ and decreases in oxygen saturation,^12^ behavioral responses such as crying and facial grimacing,^13^, ^14^ and noxious‐evoked brain activity^15^, ^16^—with activation of brain regions thought to be involved in both sensory and affective processing.^17^, ^18^ Thus, multimodal pain assessment might be best suited to capture the infant pain response.^19^

Although a number of pain scores incorporate a variety of measures derived from one or multiple systems (hereafter referred to as modalities),^20^ the relationships between modalities and their subcomponents, and the added value of including multiple measures within each modality is unclear. Behavioral measures are reported to be significantly more powerful in indicating pain than physiological measures.^21^, ^22^ However, the combination of facial expressions, cry, body movements, and physiological measures was reported to be more accurate than the individual modalities, encouraging multimodal pain assessment.^21^ This is supported by Roué and colleagues, who showed that variance in infants’ responses to venepuncture can be partially explained by two dimensions: one including behavior, salivary cortisol, and skin conductance; and one including changes in near‐infrared spectroscopy (NIRS) and physiology.^23^ Moreover, recent work by DiLorenzo and colleagues demonstrated that three items from the Neonatal Facial Coding System and one item from the Modified Behavioural Pain Scale (two widely used behavioral measures) can maintain the psychometric properties of the full scales,^24^ suggesting that the inclusion of multiple measures within a modality may not improve discrimination.

The aim of this study was to investigate the relationship between, and discriminative power of, different components of the pain response in infants aged from 34 to 42 weeks’ gestation. To this end, we used a data‐driven machine learning approach to firstly identify whether including multiple measures within the same modality improves discrimination between responses to a noxious (clinically required heel lance) and a non‐noxious stimulus (control heel lance), compared with single measures. Secondly, we investigated whether including multiple modalities improves discrimination between the responses compared with measuring single modalities.

## 2 METHODS

### 2.1 Study design

Behavioral, physiological, and neurophysiological data recorded in response to a clinically required noxious heel lance and a non‐noxious control heel lance in infants were used to build classification models to discriminate between the responses to the noxious and non‐noxious stimuli. First, models using different measures within individual modalities were assessed to ascertain whether particular measures provided better discriminative power, and whether combining measures increased classification accuracy. Next, a multimodal classification model was built. This final multimodal model was then validated in an independent test set of infants.

### 2.2 Subjects

A total of 109 infants were included in this study. Infants were recruited for a range of previously published and unpublished studies^8^, ^25^, ^26^ and were not specifically recruited for this study. Infants were recruited between 2012 and 2017 from the Maternity Ward and the Neonatal Unit at the John Radcliffe Hospital, Oxford University Hospitals NHS Foundation Trust, UK. Infants were eligible for inclusion if they were between 34 and 42 + 6 weeks’ gestation at the time of the study, were clinically stable, and were not receiving analgesics. Infants were excluded if they had an intraventricular hemorrhage greater than grade II, or other neurological abnormalities. Ethical approval was obtained from National Research Ethics Service (reference: 12/SC/0447 & 11/LO/0350). Informed written consent was obtained from parents before each study. Studies were carried out in accordance with the Declaration of Helsinki and good clinical practice guidelines.

**Figure 1:**
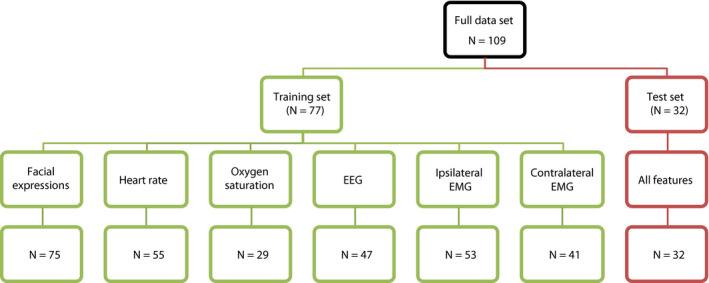
Data flowchart. Numbers indicate the number of infants with artifact‐free data recorded in each modality in the training and test sets

A training set consisting of data from 77 infants was used to investigate the use of multiple measures within individual modalities and to build the multimodal model. The multimodal model was validated in an independent test set of 32 infants. All infants in the test set had all modalities recorded without artifact following the noxious procedure, the control procedure, or both. This ensured balance in the test set and allowed for unbiased comparisons between the models. Infants where not all modalities were recorded (because the primary study which they were recruited for did not include all modalities, or because modalities were missing due to artifacts) were included in the training set (Figure 1). A machine learning approach (random forests) that can account for missing data were therefore chosen (see Random forest algorithm). Infant demographics are given in Table 1. The modalities assessed in this study were reflex withdrawal (of both the ipsilateral and contralateral limb), change in heart rate, change in oxygen saturation, facial expression responses, and noxious‐evoked brain activity recorded using EEG (electroencephalography).

**Table 1:** Infant demographics. Values given are median (25th‐75th percentile) or number (%)

	Training set (N = 77)	Test set (N = 32)
Gestational age at birth (wk)	37.0 (32.2‐40.0)	39.7 (37.1‐40.7)
Gestational age at study (wk)	38.9 (36.6‐40.3)	40.2 (37.6‐41.1)
Postnatal age at study (d)	5 (1‐23)	2 (1‐5)
Weight at birth (g)	2830 (1835‐3731)	3460 (3010‐4048)
Weight at study (g)	3045 (2143‐3768)	3460 (2955‐4048)
Sex		
Male	40 (52)	16 (50)
Female	37 (48)	16 (50)
Mode of delivery		
Spontaneous vaginal	26 (34)	12 (38)
Assisted vaginal	14 (18)	13 (41)
Caesarian section	36 (47)	7 (22)
Unknown	1 (1)	
Apgar score at 1 min	8 (6‐9)	9 (7‐10)
Apgar score at 5 min	10 (9‐10)	10 (9.5‐10)
Infant admitted to NICU	37 (48)	2 (6)

### 2.3 Experimental procedures

Clinically required heel lances were performed using a mechanical Quikheel Lancet (BD Microtainer, Becton, Dickinson and Company). Heel lances were only performed if clinically required. None of the infants were studied on more than one occasion. Following the lance, the foot was not squeezed for 30 seconds to ensure that the recorded responses were related to the noxious input from the lancet only. Before the lance, a control lance was performed by rotating a lancet by 90°, so that when released the blade did not touch the skin but other non‐noxious components of the stimulus were still present.

### 2.4 Recording techniques

#### 2.4.1 Facial expressions

Infants’ facial responses were recorded with a handheld camera for 15 seconds before and 30 seconds after the heel lance and control lance, as is the convention in the Premature Infant Pain Profile (PIPP)^27^/ Premature Infant Pain Profile—Revised (PIPP‐R) score.^28^ A LED light activated by the experimenter at the time of stimulation was used to mark the timing of the control lance or heel lance on the videos.

#### 2.4.2 Electrophysiological recordings

Electrophysiological activity from DC to 400 Hz was acquired with a SynAmps RT 64‐channel EEG system (Compumedics Neuroscan). Activity was sampled at 2000 Hz and recorded using CURRYscan7 neuroimaging suite (Compumedics Neuroscan). EEG was recorded using Ambu Neuroline disposable Ag/AgCl cup electrodes at Cz, CPz, C3, C4, FCz, Oz, T3, and T4 according to the modified international 10‐20 system, with a reference electrode at Fz and a ground electrode on the forehead. To optimize contact with the scalp, the skin was gently rubbed with EEG preparation gel (NuPrep gel; DO Weaver and Co.) prior to electrode placement and EEG conductive paste (Elefix EEG paste; Nihon Kohden) was used. Bipolar EMG electrodes (Ambu Neuroline 700 solid gel surface electrodes) on the biceps femoris of both legs were used to measure reflexes. An ECG electrode (Ambu Neuroline 700 solid gel surface electrodes) was placed on the chest and recorded with reference to Fz. Electrophysiological activity was time‐locked to the control lance and the heel lance using an accelerometer as previously described.^29^

#### 2.4.3 Oxygen saturation

Oxygen saturation was measured with a pulse oximeter placed on the infant's foot. For 16 infants, an OxiMax N‐600 pulse oximetry monitor (Nellcor) was used. For the other infants, pulse oximetry was acquired using an IntelliVue MX800 Philips patient monitor. Data were downloaded to a computer; in the case of the Philips monitor using ixTrend software (ixellence). Data were time‐locked to the stimulus using an accelerometer^29^ or by an investigator manually annotating the computer recording at the point of stimulation.

### 2.5 Analysis

#### 2.5.1 Facial expressions

Videos were epoched to include 15 seconds before the stimulus and 30 seconds afterward, and randomized so that the scorer was blinded to the stimulus type. Two researchers trained in facial expression scoring individually assessed the duration of brow bulge, eye squeeze, and nasolabial furrow in the 30 seconds after the stimulus. These facial features were chosen in accordance with the PIPP‐R score.

#### 2.5.2 ECG

ECG traces were preprocessed by extracting RR intervals as described previously.^30^ The heart rate in beats per minutes (bpm) was calculated at each second according to the mean RR interval in the previous 5 seconds. Baseline heart rate was defined as the average heart rate in the 15 seconds prestimulus. Different measures of heart rate response were compared to assess which was most discriminative between the noxious and non‐noxious stimulus. Four categories of response were considered and defined as:

- mean change: the difference between the mean heart rate in the poststimulus window and the baseline heart rate;
- maximum change: the difference between the maximum heart rate in the poststimulus window and the baseline heart rate;
- normalized mean change: the difference between the mean heart rate in the poststimulus window and the baseline heart rate, divided by the prestimulus standard deviation;
- normalized maximum change: the difference between the maximum heart rate in the poststimulus window and the baseline heart rate, divided by the prestimulus standard deviation.

Each of these values was calculated for 5, 10, 15, 20, 25, and 30 seconds windows poststimulus, giving a total of 24 different heart rate measures.

#### 2.5.3 Oxygen saturation

Similarly, a total of 24 different measures of oxygen saturation were compared. Baseline oxygen saturation was calculated as the mean in the 15 seconds prestimulus. Mean change and normalized mean change were calculated as described for heart rate. The minimum change was defined as the difference between the minimum oxygen saturation in the poststimulus window and the baseline oxygen saturation; and the normalized minimum change was defined as the difference between the minimum oxygen saturation in the poststimulus window and the baseline oxygen saturation, divided by the prestimulus standard deviation. Each of these values was calculated for 5, 10, 15, 20, 25, and 30 seconds windows poststimulus, giving a total of 24 different measures.

#### 2.5.4 EMG

EMG recordings were filtered between 10 and 500 Hz with a notch filter of 50 Hz and harmonics, and epoched from 2 seconds before the stimulus to 14.5 seconds afterward. Responses were calculated using three different approaches: the root mean square (RMS), the duration, and the amplitude.^8^ RMS was calculated in 250 ms windows after the stimulus and then the mean calculated across the first four poststimulus windows (ie, the first second after the stimulus). The duration and amplitude of the reflexes were calculated using an algorithm as previously described.^8^ Ipsilateral and contralateral recordings were assessed separately for artifacts; of the 344 available EMG recordings from either leg and to either stimulus a total of 57 were rejected, 43 due to movement artifacts in the baseline, 6 due to ECG artifacts, and 8 because no endpoint of the reflex could be identified by the algorithm.

#### 2.5.5 EEG

EEG traces were filtered between 0.5 and 70 Hz with a notch filter of 50 Hz, epoched in 1.5 second epochs, with 0.5 seconds before the stimulus, and baseline corrected to the prestimulus mean. Of the 168 available EEG traces, 16 were rejected due to movement artifacts. The magnitude of the noxious‐evoked brain activity at the Cz electrode was evaluated using three different approaches:

- A predefined validated template of noxious‐evoked brain activity.^16^ The template was projected onto individual trials in the time window 400‐700 milliseconds after the stimulus in each individual trace as previously described.^16^ This calculates a magnitude of the template of noxious‐evoked brain activity within each individual trial. To account for the age range in the study population and the associated expected variation in latency of the response, the individual traces were first Woody filtered by ±100 ms, identifying the best alignment with the template.
- Peak‐to‐peak amplitude rated by observers. Two raters assessed the presence and peak‐to‐peak amplitude of the noxious‐evoked brain activity in the responses. If the raters determined that there was no response, then the amplitude was set to 0. If the raters determined that there was a response, then they selected the negative and positive peaks and the amplitude between them was calculated. The data were first Woody filtered by ±100 ms in the region 400‐700 ms to identify the best alignment of individual trials with the group average. The data were randomized so that the raters were masked to whether the response was following a control heel lance or heel lance. For training, the raters assessed 50% of the responses together initially and then independently assessed the remaining 50% of the data. The inter‐rater agreement in identifying noxious‐evoked brain activity in the second half of the data was high (Cohen's kappa 0.77, 95% CI 0.72‐0.82, *P* < .001), and the rated response amplitude of the traces were highly correlated between raters (Pearson's *R* = .88, *P* < .001). One of the raters’ results was used in the model.
- Automated peak‐to‐peak amplitude. The negative peak in the time window 350‐450 ms and the positive peak in the time window 450‐650 ms after the stimulus were identified automatically using MATLAB, and the amplitude between them was calculated. The data were first Woody filtered by ±100 ms in the region 400‐700 ms to identify the best alignment of individual trials with the group average.

#### 2.5.6 Random forest algorithm

Since we were interested in responses that discriminate between a noxious stimulus and a non‐noxious stimulus of a similar saliency, we used classification models to distinguish between the noxious and the non‐noxious test condition. Classification models were created with the random forest algorithm,^31^ with model training and statistical analyses conducted in MATLAB (2014b, Mathworks). This approach was chosen as it is known to produce accurate predictions, to be robust to overfitting and to be modifiable to account for missing data in the training set and test set.^31^, ^32^ Furthermore, the out‐of‐bag predictions derived from the model provide an unbiased estimate of classification performance in a new data set. Optimal splitting points in decision trees were identified by calculating the impurity of the resulting nodes using the Gini diversity index.^33^ As the current study incorporates two conditions per individual, the control heel lance and the heel lance, the random forest algorithm was modified to bootstrap on subjects instead of observations, similarly to an approach proposed by Karpievitch et al.^34^

Firstly, we investigated the value of different measures within the facial expression, heart rate, oxygen saturation, reflex withdrawal, and brain activity modalities. Random forest models were trained on the duration of brow bulge, eye squeeze, and nasolabial furrow individually, and each was compared to a model based on all three expressions. Within the heart rate, oxygen saturation, and brain activity modalities, individual models were trained on each different measure and compared. Within the EMG modality, the values of EMG RMS, duration and amplitude were assessed in the ipsilateral leg and the contralateral leg separately.

In the second part of the study, a random forest model was trained on all modalities in the 61 infants from the training set for whom at least three modalities were available, using the measures with highest discrimination that were identified within the models of each individual modality. The measure used from each modality was selected based on accuracy and the area under the receiver operator characteristic (ROC) curve (AUC) (see statistical analysis), choosing the measure with higher discriminative power within our training data. However, it is important to note that most measures within individual modalities had similar discriminative power, so the measures chosen may not be the single best measure within each modality. In determining the single best measure, other factors may also be taken into account (see Discussion). The multimodal model was finally validated in the independent test set.

### 2.6 Statistical analysis

Spearman correlation coefficients were calculated to quantify correlations between measures and modalities. The performance of the random forest models was estimated by calculating the accuracy, sensitivity, specificity, and the AUC of the ROC curve. 95% confidence intervals around accuracy, sensitivity, and specificity were computed using the Wilson score interval. Accuracy, sensitivity, and specificity of different models were compared with two‐sided mid‐*P*‐value McNemar's tests.^35^ Where appropriate, models were compared to chance with the exact binomial test. ROC curves and AUC were calculated by plotting false‐positive rates against true‐positive rates for different thresholds. 95% confidence intervals for the ROC curve and the AUC were computed by 2000‐fold bootstrapping, and AUCs were compared with DeLong's test for correlated ROC curves.^36^ The out‐of‐bag predictions of the full model were stratified into a group of term infants (≥37 weeks gestation) and a group of preterm infants (<37 weeks gestation). A chi‐square test was used to compare the accuracy of the final model in each group. Exact binomial tests and chi‐square tests were performed in R3.5.2 and RStudio1.1.463. ROC curves were calculated and compared in R3.5.2 and RStudio1.1.463 using the pROC package.^37^ All other statistical analysis was conducted in MATLAB (2014b, Mathworks).

## 3 RESULTS

**Figure 2:**
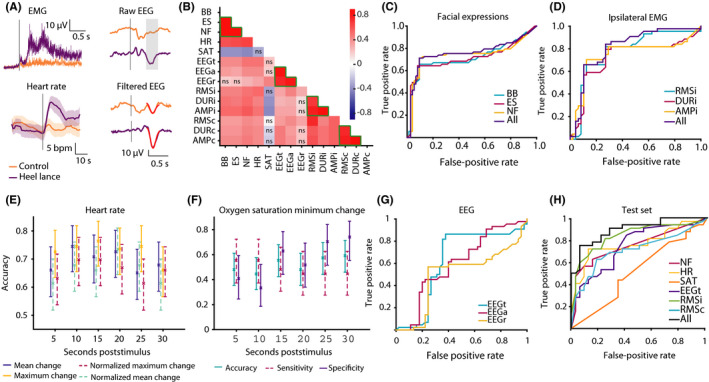
A, Average reflex withdrawal, heart rate, and EEG activity across infants in the training set in response to the control heel lance (orange) and the heel lance (purple). Average raw EEG responses are shown with the expected time window of the noxious‐evoked response shaded in gray. The (Woody) filtered EEG is shown overlaid with the template of noxious‐evoked brain activity (in red). The time of the stimulus is indicated by the gray line in each subplot. B, Correlation matrix showing Spearman's correlation coefficient between the different measures and modalities. Measures within a modality are indicated by green lines; ns indicates nonsignificant correlations (*P* ≥ .05). C‐G) Models comparing measures within individual modalities. C, ROC curves for the three individual facial expression models, and the model containing all three facial expressions. D, ROC curves for the ipsilateral reflex withdrawal models. E, Accuracy of the 24 different heart rate models. Error bars represent 95% confidence intervals. F, Accuracy, sensitivity, and specificity of the oxygen saturation minimum change (difference between the minimum oxygen saturation in the poststimulus window and the mean baseline oxygen saturation) models at 6 time points. G, ROC curves of the models measuring noxious‐evoked brain activity. H, Multimodal model; ROC curves showing the performance of the individual and the full model on the test set. (Abbreviations: BB, Brow bulge duration; ES, eye squeeze duration; NF, nasolabial furrow duration; EEGt, magnitude of the noxious‐evoked response measured with the validated template of noxious‐evoked activity; EEGa, automated peak‐to‐peak calculation; EEGr, magnitude of noxious‐evoked potential assessed by raters; HR, heart rate change from prestimulus mean to maximum in 15 s poststimulus; SAT, oxygen saturation change from prestimulus mean to minimum in 30 s poststimulus; RMSi, root mean square of ipsilateral reflex withdrawal; DURi, duration of ipsilateral reflex withdrawal response; AMPi, amplitude of ipsilateral reflex withdrawal response, and similarly for the contralateral leg—RMSc, DURc, AMPc.)

Multimodal responses to a noxious and non‐noxious (clinically required heel lance and control heel lance) were recorded in 109 infants from 34 to 42 weeks’ gestational age at the time of study (Figure 2A).

### 3.1 Assessing different measures within individual modalities

The first part of this study investigated the relationships between measures within each modality by determining whether multiple measures in the same modality could improve discrimination between the noxious and non‐noxious stimuli, compared with single measures, and whether any single measure performed better than other measures. We compared model accuracy, specificity, sensitivity, and AUC. Statistical comparisons were calculated as a guide, but should not be interpreted as clearly indicating the best option as this is context dependent. For example, in some situations it may be more important to have a model with higher specificity or sensitivity or a balance between the two. Consequently, this may mean a different feature to the one selected here is more appropriate in different contexts. We constructed models for each of facial expression, heart rate, oxygen saturation, reflex withdrawal, and noxious‐evoked brain activity in turn.

### 3.2 Facial expression

Brow bulge, eye squeeze, and nasolabial furrow duration were highly correlated (Figure 2B). The three facial expressions individually could discriminate similarly well between the noxious and the non‐noxious conditions and combining all three did not yield higher accuracy, sensitivity, specificity, or AUC (Figure 2C, Table S1). This demonstrates that combining these three measures of facial expression did not improve discrimination between the noxious and the non‐noxious stimulus over single measures. Nasolabial furrow had the highest accuracy and AUC of the individual measures. As this feature was the measure with highest discriminative power, it was selected for use in the multimodal model.

### 3.3 Reflex withdrawal

Reflex withdrawal was quantified by considering the duration, amplitude, and RMS of the reflexes of the legs ipsilateral and contralateral to the site of stimulation (see Methods). For both legs, these measures were significantly correlated (Figure 2B). Combining the three measures of reflex withdrawal did not improve discrimination between the noxious and non‐noxious stimuli over the single measure of RMS (Figure 2D, Table S1). Although the ipsilateral and contralateral RMS were well correlated, the ipsilateral duration and amplitude of the reflexes were less well correlated with the contralateral EMG measures (Figure 2B). Both ipsilateral and contralateral responses were included in the full multimodal model, as they are likely to provide added discriminative value.^7^

### 3.4 Physiology

For heart rate and oxygen saturation, we examined whether any single measure of change performed better than others. Four different measures of heart rate (mean change, maximum change, normalized mean change, and normalized maximum change) were considered across six different time windows, giving a total of 24 models (see Methods). The maximum change (the difference between the prestimulus mean and the poststimulus peak) in the 15 seconds poststimulus had the highest accuracy and the third highest AUC of the 24 models (Figure 2E, Table S2), consistent with the steep rise in heart rate in the first 15 seconds after the stimulus (Figure 2A). Therefore, we selected the maximum change in heart rate in the 15 seconds poststimulus for inclusion in the final multimodal model.

Oxygen saturation provided poor discrimination, as it was not significantly different to the 50% threshold that would be expected solely based on chance (Table S3). Nevertheless, the decrease in oxygen saturation from baseline to the 30 seconds poststimulus (part of the PIPP‐R score) had a relatively high specificity of 74% (Figure 2F, Table S3), highlighting that when a decrease in oxygen saturation occurs it is likely indicative of the noxious stimulus.

### 3.5 EEG

Assessment of the magnitude of noxious‐evoked brain activity was compared using three different approaches: (a) using a validated template 16; (b) using automated peak‐to‐peak amplitude detection software and (c) observer ratings of amplitude (see Methods for further details). The magnitude of noxious‐evoked brain activity assessed by the validated template was well correlated with automated peak‐to‐peak amplitude, but less well correlated with observer ratings of amplitude (Figure 2B). Using the validated template of noxious‐evoked brain activity was significantly more accurate than automated peak‐to‐peak amplitude calculations (Table S4) and had a significantly higher AUC than observer ratings (Figure 2G, Table S4). These findings, combined with the fact that the template provides an objective measure, which cannot be biased by an observer and does not require expert ratings of the responses, demonstrates the appropriateness of using the template.

### 3.6 Relationships in a multimodal classification model

In the second part of the study, we wanted to address the relationships *between* modalities. Overall, correlations between modalities were less strong than correlations within modalities (Figure 2B). Since all modalities, except oxygen saturation, had reasonable accuracy (>70%) in discriminating responses, it is possible that the different modalities explain distinct components of the variance in the evoked responses to the two stimuli. This would imply that combining measures would lead to higher discriminative power.

To explore this hypothesis, a final model containing the “best” individual measure from each modality was trained on 61 infants (Table 2) and validated in an independent test set of 32 infants (Table 3). In the training set (out‐of‐bag predictions), model accuracy was 0.81 (confidence interval 0.72‐0.87). To test whether discriminative power was affected by age, we divided the training set into preterm (34‐36 weeks gestational age) and term (37‐42 weeks gestational age) infants. There was no significant difference in accuracy at discriminating between noxious and non‐noxious stimuli between the two groups (chi‐square test, *P* = .22, Table 2). In the test set, the accuracy of the full model was also 0.81 (confidence interval 0.70‐0.89) and the AUC was 0.90 (confidence interval: 0.78‐0.95) signifying a good balance between sensitivity and specificity at all thresholds. This was significantly higher than the AUC for the individual models of nasolabial furrow, heart rate, oxygen saturation, EEG, and contralateral EMG and higher than the AUC for the ipsilateral EMG (Figure 2H, Table 3). This indicates that including multiple modalities improves discrimination between the noxious and non‐noxious responses. Moreover, the full model had a significantly higher AUC than a model with only behavioral and physiological responses (nasolabial furrow, heart rate change, and oxygen saturation change; Table 3). This suggests that discrimination between noxious and non‐noxious stimuli is improved with the addition of the neurophysiological measures.

**Table 2:** Model metrics for the full model, obtained in the training set

		Model metrics (out‐of‐bag)			
		Accuracy	Sensitivity	Specificity	AUC
Full model (61 infants, of which 40 term, 21 preterm, 118 observations)	All ages	0.81 (0.72‐0.87)	0.76 (0.64‐0.85)	0.85 (0.73‐0.92)	0.89 (0.81‐0.94)
	Preterm (<37 wk)	0.88 (0.74‐0.95)	0.80 (0.58‐0.92)	0.95 (0.77‐0.99)	0.94 (0.81‐0.99)
	Term (≥37 wk)	0.77 (0.66‐0.85)	0.74 (0.59‐0.85)	0.79 (0.64‐0.89)	0.86 (0.75‐0.93)

Out‐of‐bag accuracy, sensitivity, specificity, and AUC (area under the ROC curve) are reported along with confidence intervals in brackets.

**Table 3:** Model metrics and statistics for the individual models and the full model, obtained in the test set

		Model metrics (test set)				*P*‐Values (comparison to full model)	
		Accuracy	Sensitivity	Specificity	AUC	Accuracy	AUC
Model	Nasolabial furrow	0.70 (0.58‐0.80)	0.56 (0.39‐0.72)	0.84 (0.68‐0.93)	0.76 (0.64‐0.85)	.022*	.00079*
	Heart rate	0.73 (0.62‐0.83)	0.72 (0.55‐0.84)	0.75 (0.58‐0.87)	0.77 (0.63‐0.87)	.21	.027*
	Oxygen saturation	0.55 (0.43‐0.66)	0.44 (0.28‐0.61)	0.66 (0.48‐0.80)	0.52 (0.38‐0.65)	.00091*	<.0001*
	EEG	0.64 (0.52‐0.75)	0.59 (0.42‐0.74)	0.69 (0.51‐0.82)	0.75 (0.60‐0.85)	.012*	.013*
	Ipsilateral EMG	0.77 (0.65‐0.85)	0.81 (0.65‐0.91)	0.72 (0.55‐0.84)	0.84 (0.71‐0.92)	.50	.34
	Contralateral EMG	0.72 (0.60‐0.81)	0.63 (0.45‐0.77)	0.81 (0.65‐0.91)	0.71 (0.56‐0.82)	.21	.0081*
	Facial, HR, saturation	0.75 (0.63‐0.84)	0.75 (0.58‐0.87)	0.75 (0.58‐0.87)	0.80 (0.65‐0.89)	.13	.0069*
	Full	0.81 (0.70‐0.89)	0.78 (0.61‐0.89)	0.84 (0.68‐0.93)	0.90 (0.78‐0.95)	N/A	N/A

Note

Models of individual measures were compared with the full model. A model combining measures of facial expression (nasolabial furrow), heart rate and oxygen saturation was also compared with the full model. Accuracy, sensitivity, specificity, and AUC (area under the ROC curve) in the test set are reported along with confidence intervals in brackets. *P*‐values are given for the comparison between the accuracy of the individual models and the full model (mid‐*P*‐value McNemar's test) and between the AUC’s of the individual models and the full model (DeLong's test).

* Indicates *P* < .05.

## 4 DISCUSSION

In this study, we investigated the multimodal relationships in responses to noxious and non‐noxious stimuli in infants aged 34 to 42 + 6 weeks at the time of study. We quantified noxious‐evoked changes in infant behavior, physiology, reflex withdrawal activity, and brain activity and used accuracy of discrimination between a noxious and non‐noxious stimulus as a marker to establish the benefit of including both multiple modalities and multiple measures within each modality. Random forests machine learning provided a data‐driven approach to assess classification accuracy across different models,^31^ identifying discriminative patterns first within a training set and then validating these patterns within a test set.^38^ The models identify features within the data that can predict whether responses are evoked by the noxious or non‐noxious stimuli. Validating the model in an independent test set gives an accurate picture as to how the model will perform with any independent data. We demonstrate that measures within modalities (eg, multiple measures of facial grimacing) are highly correlated and that single measures perform as well as multiple measures. In contrast, measures recorded across different modalities are less correlated, and as such, including different modalities improves the discrimination between a noxious and non‐noxious stimulus. This indicates that there is a benefit to including activity recorded across a range of modalities in infant pain assessment, which is consistent with recommendations of other authors examining multimodal responses to pain.^21^, ^23^

Overall, we find strong relationships between measures within modalities and consequently including multiple measures within a modality does not improve discrimination compared with using a single measure of reflex withdrawal or facial expression. Nevertheless, although we found, for example, that the duration of nasolabial furrow alone had similar discriminative accuracy compared with the combination of all three facial expressions, this should not be interpreted to suggest that nasolabial furrow will always be the best facial expression measure to use. Other measures might be more appropriate in different contexts; for instance, in ventilated infants, where the nose and mouth are obscured from view, brow bulge, or eye squeeze could be assessed. In this study, *P*‐values were used only as a guide to select the best measure for inclusion in our final multimodal model. Many infant pain assessment tools make use of multiple measures within a single modality, and while these data do not imply that existing pain scores should be modified for use in pain assessment (as these are validated constructs), it is important to note that multiple measures within a single modality may not always add value.

Correlations between measures that span different modalities were considerably less strong than the correlations observed within modalities, and the discriminative power of the model with multiple modalities was higher than the models with individual modalities. This highlights the benefit of using multimodal assessment when attempting to quantify infant pain experience. Behavioral and physiological measures of pain are frequently combined in infant pain assessment tools.^5^, ^20^ For example, the well‐validated PIPP‐R combines scores from three different facial components, change in heart rate, and change in the oxygen saturation, as well as baseline behavioral state and gestational age.^28^ We find that neurophysiological measures are only moderately correlated to the behavioral and physiological modalities, implying that noxious‐evoked brain activity and reflex withdrawal measure different components of the infant's experience, and demonstrating the value of including neurophysiological measures in infant pain assessment.

As pain is a subjective experience and necessarily involves cortical activity,^39^ measuring brain activity can provide important insight into how nociceptive input is processed by the brain. However, the template of noxious‐evoked brain activity that has previously been derived is limited as it does not measure all nociceptive activity that takes place across the infant's brain.^16^ Furthermore, the sensitivity and specificity of this measure for individual infants was previously noted to be only moderate (approximately 65%).^16^ While developing a validated signature of noxious‐evoked brain activity which incorporates other features (such as the data acquired from fMRI studies 17, 40) will add additional information, inclusion of noxious‐evoked brain activity in the full multimodal model in this study (measured using the validated template) provided increased power to discriminate noxious from non‐noxious procedures.

Some studies have questioned the utility of physiological measures in infant pain assessment due to their lack of specificity to noxious stimulation.^9^ Välitalo and colleagues used a graded response model to examine individual components of the COMFORT and PIPP scales and found that behavioral measures were more informative than physiological measures.^22^ However, here we find that physiological measures add value. In particular, while oxygen saturation has low sensitivity, it has moderately high specificity. Moreover, using a poststimulus time window of 15 seconds (shorter than the 30 seconds used in the PIPP‐R score) improved discrimination based on change in heart rate. While this time window may not be appropriate for longer procedures, and consideration of other non‐noxious control stimuli is needed, using a data‐driven approach such as the one used here may identify features within physiological responses which are accurate surrogate measures of infant pain. Moreover, in the case of clinical trials of analgesics, including multiple modalities is beneficial in the assessment of safety, as well as efficacy, of a drug.^41^, ^42^

Here, we considered responses to a clinically required heel lance and compared them to responses to a non‐noxious control stimulus. These stimuli were closely matched, allowing us to identify features that could discriminate the noxious component of the procedure. However, the models’ ability to discriminate these stimuli with relatively high accuracy does not mean that this would be directly applicable to other noxious and non‐noxious procedures which may vary in intensity and duration. The specific model produced here was not designed to develop a pain score but rather to investigate the relationship between different measures and modalities within infant pain assessment. Indeed, if these data were to be used to aid in the development of a new pain measurement tool, the importance of testing other features, such as the ability for such a tool to detect changes in pain intensity after a pain‐reducing intervention has recently been eloquently highlighted.^43^

Using the classification approach taken here, a misclassified response is counted as a failure of the model. Ultimately, this is important, as we would strive to achieve a model that can identify whether an infant is in pain with 100% accuracy. However, it is currently unclear what it means when an infant does not mount a response to a noxious stimulus in any given modality.^44^ For example, infants can fail to respond behaviorally 25 and it is not well understood whether this truly reflects a lack of pain experience or is driven by other reasons such as lethargy.^44^, ^45^ It is possible that a lack of response across several modalities, which would lead to misclassification in our model, is because the infant does not find the stimulus painful. However, interpreting these data are complex and further research is warranted.

In this study, we considered infants from 34 to 42 weeks’ gestation at the time of study. Infants younger than this are more likely to display nonmodality‐specific brain activity responses,^46^ reflex to tactile as well as noxious stimuli,^6^ and display facial grimacing to both noxious and non‐noxious stimuli.^25^ Moreover, gestational age is a strong influential factor in creating automated pain assessment approaches 47 and responses to noxious stimulation increase over the age range studied here.^6^, ^8^, ^25^ While we did not see a difference in accuracy of the multimodal model between preterm and term infants, an important extension of this work will be to determine how well multiple assessment modalities can discriminate between noxious and non‐noxious stimuli in younger preterm infants. Deriving an age‐dependent multimodal pain assessment tool for preterm infants is critical given the high burden of pain experienced during the course of neonatal treatment. Moreover, many other factors may influence infants’ responses to noxious stimuli, including sleep state, mode of delivery, current medication, pathology, and type and timing of feeding.^48^, ^49^ Investigating how discrimination between noxious and non‐noxious stimuli is influenced by these factors will shed further light on the infant pain experience.

In summary, we demonstrate that the inclusion of data recorded across multiple modalities in the assessment of infant pain (including pain‐related changes in behavior, reflex withdrawal, physiology, and brain activity) improves our ability to discriminate infants’ responses to noxious and innocuous stimuli. In contrast, inclusion of data acquired from taking multiple measures within a single modality (eg, alternative measures of facial grimacing) does not improve discrimination between noxious and innocuous stimuli. This is particularly relevant in the research setting where combining neurophysiological, physiological, and behavioral measures can provide better insight into the infant pain experience and lead to a better understanding of how analgesic interventions influence nociceptive processing across all levels of the central nervous system.

## CONFLICT OF INTEREST

The authors declare no conflicts of interest.
