# Supplementary material for: Multimodal pain assessment improves discrimination between noxious and non‐noxious stimuli in infants
Source: Paediatr Neonatal Pain. 2019 Sep 9;1(1):21–30. doi: 10.1002/pne2.12007 (PMC8974881; doi:10.1002/pne2.12007)
Supplement: Supplementary file 1 [file PNE2-1-21-s001.docx]

**Supplementary material**

**Multimodal pain assessment improves discrimination between noxious and non-noxious stimuli in infants**

Marianne van der Vaart, Eugene Duff, Nader Raafat, Richard Rogers, Caroline Hartley, Rebeccah Slater

|  |  | **Model metrics** | | | | **P-Values** | | | |
| --- | --- | --- | --- | --- | --- | --- | --- | --- | --- |
|  |  | **Accuracy** | **Sensitivity** | **Specificity** | **AUC** | Brow bulge | Eye squeeze | Nasolabial furrow | Combined |
| **Facial expression**  (75 infants, 147 observations) | Brow bulge | 0.78  (0.71-0.84) | 0.66  (0.54-0.76) | 0.91  (0.82-0.95) | 0.72  (0.63-0.80) | N/A | 0.81 | 0.55 | 0.20 |
|  | Eye squeeze | 0.77  (0.69-0.83) | 0.62  (0.50-0.72) | 0.92  (0.83-0.96) | 0.71  (0.62-0.80) | 0.38 | N/A | 0.40 | 0.085 |
|  | Nasolabial furrow | 0.80  (0.73-0.86) | 0.71  (0.60-0.80) | 0.89  (0.80-0.94) | 0.74  (0.64-0.82) | 0.29 | 0.11 | N/A | 0.39 |
|  | Combined | 0.80  (0.72-0.85) | 0.68  (0.57-0.78) | 0.91  (0.82-0.95) | 0.76  (0.66-0.83) | 0.38 | 0.13 | 0.63 | N/A |
|  |  |  |  |  |  | RMS | Duration | Amplitude | Combined |
| **Ipsilateral reflex withdrawal**  (53 infants, 92 observations) | RMS | 0.72  (0.62-0.80) | 0.70  (0.56-0.82) | 0.73  (0.59-0.83) | 0.78  (0.66-0.87) | N/A | 0.36 | 0.58 | 0.31 |
|  | Duration | 0.75  (0.65-0.83) | 0.77  (0.63-0.87) | 0.73  (0.59-0.83) | 0.72  (0.59-0.82) | 0.48 | N/A | 0.37 | **0.031*** |
|  | Amplitude | 0.78  (0.69-0.85) | 0.68  (0.53-0.80) | 0.88  (0.75-0.94) | 0.75  (0.62-0.84) | 0.17 | 0.42 | N/A | 0.067 |
|  | Combined | 0.73  (0.63-0.81) | 0.70  (0.56-0.82) | 0.75  (0.61-0.85) | 0.81  (0.71-0.89) | 0.75 | 0.55 | 0.11 | N/A |
|  |  |  |  |  |  | RMS | Duration | Amplitude | Combined |
| **Contralateral reflex withdrawal**  (41 infants, 76 observations) | RMS | 0.72  (0.61-0.81) | 0.65  (0.49-0.78) | 0.79  (0.64-0.89) | 0.76  (0.63-0.86) | N/A | 0.31 | 0.53 | 0.80 |
|  | Duration | 0.78  (0.67-0.86) | 0.73  (0.57-0.85) | 0.82  (0.67-0.91) | 0.71  (0.55-0.82) | 0.27 | N/A | 0.26 | 0.096 |
|  | Amplitude | 0.75  (0.64-0.83) | 0.70  (0.54-0.83) | 0.79  (0.64-0.89) | 0.73  (0.60-0.84) | 0.58 | 0.38 | N/A | 0.25 |
|  | Combined | 0.76  (0.66-0.84) | 0.73  (0.57-0.85) | 0.79  (0.64-0.89) | 0.77  (0.63-0.87) | 0.42 | 0.50 | 0.63 | N/A |

**Supplementary table 1: Model metrics and statistics for facial expression and reflex withdrawal.** Models of individual measures were compared with a combined model, which included all measures for that modality. Out-of-bag accuracy, sensitivity, specificity and AUC (area under the ROC curve) are reported along with confidence intervals in brackets. P-values below the diagonal compare model accuracies (mid-p value McNemar’s test) and above the diagonal compare model AUC’s (DeLong’s test). The number of observations indicates response to the control heel lance and heel lance included in each model. RMS: root mean square. * indicates p < 0.05. Green shading indicates measure used in full multimodal model.

|  |  | **Model metrics** | | | |
| --- | --- | --- | --- | --- | --- |
|  |  | **Accuracy** | **Sensitivity** | **Specificity** | **AUC** |
| **Heart rate (mean change)**  55 infants (106 observations) | **0-5** | 0.66  (0.57-0.74) | 0.55  (0.41-0.67) | 0.77  (0.64-0.87) | 0.62  (0.51-0.73) |
|  | **0-10** | 0.75  (0.65-0.82) | 0.55  (0.41-0.67) | 0.94  (0.85-0.98) | 0.65  (0.54-0.76) |
|  | **0-15** | 0.71  (0.61-0.79) | 0.72  (0.58-0.82) | 0.70  (0.56-0.80) | 0.71  (0.60-0.80) |
|  | **0-20** | 0.73  (0.63-0.80) | 0.72  (0.58-0.82) | 0.74  (0.60-0.84) | 0.78  (0.67-0.86) |
|  | **0-25** | 0.65  (0.56-0.73) | 0.68  (0.55-0.79) | 0.62  (0.49-0.74) | 0.66  (0.55-0.76) |
|  | **0-30** | 0.68  (0.59-0.76) | 0.60  (0.47-0.72) | 0.75  (0.62-0.85) | 0.61  (0.49-0.71) |
| **Heart rate (normalised mean change)** | **0-5** | 0.61  (0.52-0.70) | 0.58  (0.45-0.71) | 0.64  (0.51-0.76) | 0.61  (0.49-0.71) |
|  | **0-10** | 0.68  (0.59-0.76) | 0.70  (0.56-0.80) | 0.66  (0.53-0.77) | 0.65  (0.54-0.75) |
|  | **0-15** | 0.66  (0.57-0.74) | 0.66  (0.53-0.77) | 0.66  (0.53-0.77) | 0.75  (0.64-0.83) |
|  | **0-20** | 0.74  (0.64-0.81) | 0.79  (0.67-0.88) | 0.68  (0.55-0.79) | 0.73  (0.63-0.82) |
|  | **0-25** | 0.68  (0.59-0.76) | 0.74  (0.60-0.84) | 0.62  (0.49-0.74)_ | 0.65  (0.53-0.75) |
|  | **0-30** | 0.61  (0.52-0.70) | 0.53  (0.40-0.66) | 0.70  (0.56-0.80) | 0.54  (0.42-0.65) |
| **Heart rate (maximum change)** | **0-5** | 0.73  (0.63-0.80) | 0.58  (0.45-0.71) | 0.87  (0.75-0.93) | 0.68  (0.57-0.79) |
|  | **0-10** | 0.75  (0.65-0.82) | 0.75  (0.62-0.85) | 0.74  (0.60-0.84) | 0.77  (0.67-0.86) |
|  | **0-15** | 0.76  (0.67-0.83) | 0.74  (0.60-0.84) | 0.79  (0.67-0.88) | 0.77  (0.66-0.85) |
|  | **0-20** | 0.74  (0.64-0.81) | 0.74  (0.60-0.84) | 0.74  (0.60-0.84) | 0.74  (0.62-0.83) |
|  | **0-25** | 0.75  (0.65-0.82) | 0.74  (0.60-0.84) | 0.75  (0.62-0.85) | 0.68  (0.56-0.79) |
|  | **0-30** | 0.66  (0.57-0.74) | 0.62  (0.49-0.74) | 0.70  (0.56-0.80) | 0.70  (0.58-0.80) |
| **Heart rate (normalised maximum change)** | **0-5** | 0.63  (0.54-0.72) | 0.62  (0.49-0.74) | 0.64  (0.51-0.76) | 0.64  (0.53-0.74) |
|  | **0-10** | 0.70  (0.61-0.78) | 0.75  (0.62-0.85) | 0.64  (0.51-0.76) | 0.70  (0.58-0.80) |
|  | **0-15** | 0.70  (0.61-0.78) | 0.66  (0.53-0.77) | 0.74  (0.60-0.84) | 0.70  (0.59-0.80) |
|  | **0-20** | 0.67  (0.58-0.75) | 0.64  (0.51-0.76) | 0.70  (0.56-0.80) | 0.70  (0.58-0.79) |
|  | **0-25** | 0.61  (0.52-0.70) | 0.66  (0.53-0.77) | 0.57  (0.43-0.69) | 0.65  (0.53-0.75) |
|  | **0-30** | 0.66  (0.57-0.74) | 0.66  (0.53-0.77) | 0.66  (0.53-0.77) | 0.68  (0.56-0.77) |

|  |  | **P-values** | | | | | |
| --- | --- | --- | --- | --- | --- | --- | --- |
|  |  | **0-5** | **0-10** | **0-15** | **0-20** | **0-25** | **0-30** |
| **Heart rate (maximum change)** | **0-5** | N/A | 0.081 | 0.084 | 0.34 | 1 | 0.76 |
|  | **0-10** | 0.65 | N/A | 0.97 | 0.13 | **0.0019*** | **0.040*** |
|  | **0-15** | 0.33 | 0.38 | N/A | 0.13 | **0.0034*** | **0.034*** |
|  | **0-20** | 0.81 | 0.69 | 0.13 | N/A | **0.029*** | 0.16 |
|  | **0-25** | 0.66 | 1 | 0.45 | 0.73 | N/A | 0.47 |
|  | **0-30** | 0.17 | **0.021*** | **0.0042*** | **0.035*** | **0.013*** | N/A |

**Supplementary table 2: Model metrics and statistics for heart rate.** Out-of-bag accuracy, sensitivity, specificity and AUC (area under the ROC curve) are reported along with confidence intervals in brackets for the 24 different models (see Methods). P-values below the diagonal compare model accuracies (mid-p value McNemar’s test) and above the diagonal compare model AUC’s (DeLong’s test) for the 6 maximum change models. * indicates p < 0.05. Green shading indicates measure used in full multimodal model.

|  |  | **Model metrics** | | | |
| --- | --- | --- | --- | --- | --- |
|  | **Time window** | **Accuracy** | **Sensitivity** | **Specificity** | **AUC** |
| **Oxygen saturation (mean change)**  29 infants, 54 observations | **0-5** | 0.48  (0.35-0.61) | 0.56  (0.37-0.72) | 0.41  (0.25-0.59) | 0.34  (0.20-0.51) |
|  | **0-10** | 0.52  (0.39-0.65) | 0.56  (0.37-0.72) | 0.48  (0.31-0.66) | 0.37  (0.22-0.52) |
|  | **0-15** | 0.44  (0.32 -0.58) | 0.41  (0.25-0.59) | 0.48  (0.31-0.66) | 0.32  (0.18 -0.48) |
|  | **0-20** | 0.48  (0.35-0.61) | 0.44  (0.28-0.63) | 0.52  (0.34-0.69) | 0.37  (0.23-0.54) |
|  | **0-25** | 0.54  (0.41-0.66) | 0.56  (0.37-0.72) | 0.52  (0.34-0.69) | 0.42  (0.27-0.59) |
|  | **0-30** | 0.57  (0.44-0.70) | 0.52  (0.34-0.69) | 0.63  (0.44-0.78) | 0.47  (0.32-0.63) |
| **Oxygen saturation (normalised mean change** | **0-5** | 0.48  (0.35-0.61) | 0.52  (0.33-0.70) | 0.44  (0.27-0.63) | 0.39  (0.24-0.56) |
|  | **0-10** | 0.52  (0.39-0.65) | 0.56  (0.37-0.73) | 0.48  (0.30-0.67) | 0.37  (0.23-0.54) |
|  | **0-15** | 0.39  (0.27-0.53) | 0.16  (0.06-0.35) | 0.62  (0.43-0.78) | 0.35  (0.20-0.51) |
|  | **0-20** | 0.46  (0.33-0.59) | 0.080  (0.02-0.25) | 0.81  (0.63-0.92) | 0.31  (0.17-0.47) |
|  | **0-25** | 0.52  (0.39-0.65) | 0  (0.0-0.13) | 1  (0.88-1.0) | 0.39  (0.25-0.56) |
|  | **0-30** | 0.47  (0.34-0.60) | 0.23  (0.11-0.42) | 0.70  (0.52-0.84) | 0.48  (0.33-0.64) |
| **Oxygen saturation (minimum change)** | **0-5** | 0.48  (0.35-0.61) | 0.56  (0.37-0.72) | 0.41  (0.25-0.59) | 0.47  (0.32-0.63) |
|  | **0-10** | 0.44  (0.32-0.58) | 0.56  (0.37-0.72) | 0.33  (0.19-0.52) | 0.53  (0.35-0.68) |
|  | **0-15** | 0.56  (0.42-0.68) | 0.48  (0.31-0.66) | 0.63  (0.44-0.78) | 0.46  **(**0.30-0.63) |
|  | **0-20** | 0.48  (0.35-0.61) | 0.44  (0.28-0.63) | 0.52  (0.34-0.69) | 0.53  (0.37-0.69) |
|  | **0-25** | 0.57  (0.44-0.70) | 0.44  (0.28-0.63) | 0.70  (0.52-0.84) | 0.43  (0.28-0.60) |
|  | **0-30** | 0.59  (0.46-0.71) | 0.44  (0.28-0.63) | 0.74  (0.55-0.87) | 0.47  (0.32-0.64) |
| **Oxygen saturation (normalised minimum change)** | **0-5** | 0.38  (0.26-0.52) | 0.44  (0.27-0.63) | 0.32  (0.17-0.52) | 0.24  (0.12-0.39) |
|  | **0-10** | 0.44  (0.31-0.58) | 0.44  (0.27-0.63) | 0.44  (0.27-0.63) | 0.40  (0.25-0.57) |
|  | **0-15** | 0.38  (0.26-0.52) | 0.44  (0.27-0.63) | 0.32  (0.17-0.52) | 0.33  (0.20-0.51) |
|  | **0-20** | 0.46  (0.33-0.60) | 0.36  (0.20-0.55) | 0.56  (0.37-0.73) | 0.43  (0.28-0.61) |
|  | **0-25** | 0.42  (0.29-0.56) | 0.40  (0.23-0.59) | 0.44  (0.27-0.63) | 0.33  (0.19-0.50 |
|  | **0-30** | 0.48  (0.35-0.61) | 0.36  (0.20-0.55) | 0.60  (0.41-0.77) | 0.40  (0.24-0.58) |

|  |  | **P-values** | | | | | |
| --- | --- | --- | --- | --- | --- | --- | --- |
|  |  | **0-5** | **0-10** | **0-15** | **0-20** | **0-25** | **0-30** |
| **Saturation (minimum change)** | **0-5** | N/A | 0.50 | 0.96 | 0.50 | 0.69 | 0.97 |
|  | **0-10** | 0.65 | N/A | 0.13 | 0.93 | 0.15 | 0.49 |
|  | **0-15** | 0.30 | 0.092 | N/A | 0.29 | 0.57 | 0.90 |
|  | **0-20** | 1 | 0.58 | 0.30 | N/A | **0.015*** | 0.32 |
|  | **0-25** | 0.31 | 0.12 | 0.75 | 0.11 | N/A | 0.31 |
|  | **0-30** | 0.21 | 0.064 | 0.55 | **0.039*** | 0.50 | N/A |
|  | **Comparison to chance** | 0.89 | 0.50 | 0.50 | 0.89 | 0.34 | 0.22 |

**Supplementary table 3: Model metrics and statistics for oxygen saturation.** Out-of-bag accuracy, sensitivity, specificity and AUC (area under the ROC curve) are reported along with confidence intervals in brackets for the 24 different models (see Methods). P-values below the diagonal compare model accuracies (mid-p value McNemar’s test) and above the diagonal compare model AUC’s (DeLong’s test) for the 6 minimum change models. Bottom line shows comparison to chance. * indicates p < 0.05. Green shading indicates measure used in full multimodal model.

|  |  |  |  |  |  |  |  |  |
| --- | --- | --- | --- | --- | --- | --- | --- | --- |
|  |  | **Model metrics** | | | | **P-values** | | |
|  |  | Accuracy | Sensitivity | Specificity | AUC | Template | Automated | Ratings |
| **EEG**  (47 infants, 89 observations) | Template | 0.73  (0.63-0.81) | 0.82  (0.68-0.90) | 0.64  (0.50-0.77) | 0.62  (0.49-0.75) | N/A | 0.67 | **0.049*** |
|  | Automated | 0.57  (0.47-0.67) | 0.55  (0.40-0.68) | 0.60  (0.45-0.73) | 0.60  (0.46-0.71) | **0.0026*** | N/A | 0.059 |
|  | Ratings | 0.66  (0.56-0.75) | 0.57  (0.42-0.70) | 0.76  (0.61-0.86) | 0.50  (0.38-0.63) | 0.21 | 0.078 | N/A |
|  |  |  |  |  |  |  |  |  |

**Supplementary table 4: Model metrics and statistics for EEG measures.** Out-of-bag accuracy, sensitivity, specificity and AUC (area under the ROC curve) with confidence intervals in brackets are given for the models of each individual EEG measure. P-values below the diagonal compare model accuracies (mid-p value McNemar’s test) and above the diagonal compare model AUC’s (DeLong’s test). The number of observations indicates response to the control heel lance and heel lance included in each model. * indicates p < 0.05. Green shading indicates measure used in full multimodal model.
